# Diffusion kurtosis imaging and dynamic contrast-enhanced MRI for the differentiation of parotid gland tumors

**DOI:** 10.1007/s00330-021-08312-y

**Published:** 2021-10-12

**Authors:** Nan Huang, Yu Chen, Dejun She, Zhen Xing, Tanhui Chen, Dairong Cao

**Affiliations:** 1 Department of Radiology, The First Affiliated Hospital of Fujian Medical University, 20 Cha-Zhong Road, Fuzhou, Fujian 350005 People’s Republic of China; 2 Department of Radiology, Fujian Key Laboratory of Precision Medicine for Cancer, the First Affiliated Hospital, Fujian Medical University, Fuzhou, 350005 Fujian China; 3 Key Laboratory of Radiation Biology of Fujian Higher Education Institutions, the First Affiliated Hospital, Fujian Medical University, Fuzhou, 350005 Fujian China

**Keywords:** Parotid gland, Parotid neoplasms, Magnetic resonance imaging, Diffusion, Perfusion

## Abstract

**Objective:**

To assess the usefulness of combined diffusion kurtosis imaging (DKI) and dynamic contrast-enhanced MRI (DCE-MRI) in the differentiation of parotid gland tumors.

**Methods:**

Seventy patients with 80 parotid gland tumors who underwent DKI and DCE-MRI were retrospectively enrolled and divided into four groups: pleomorphic adenomas (PAs), Warthin tumors (WTs), other benign tumors (OBTs), and malignant tumors (MTs). DCE-MRI and DKI quantitative parameters were measured. The Kruskal–Wallis H test and post hoc test with Bonferroni correction and ROC curve were used for statistical analysis.

**Results:**

WTs demonstrated the highest *K*_ep_ value (median 1.89, interquartile range [1.46–2.31] min^−1^) but lowest *V*_e_ value (0.20, [0.15–0.25]) compared with PAs (*K*_ep_, 0.34 [0.21–0.55] min^−1^; *V*_e_, 0.36 [0.24–0.43]), OBTs (*K*_ep_, 1.22 [0.27–1.67] min^−1^; *V*_e_, 0.28 [0.25–0.41]), and MTs (*K*_ep_, 0.71 [0.50–1.23] min^−1^; *V*_e_, 0.35 [0.26–0.45]) (all *p* < .05). MTs had the lower *D* value (1.10, [0.88–1.29] × 10^−3^ mm^2^/s) compared with PAs (1.81, [1.60–2.20] × 10^−3^ mm^2^/s) and OBTs (1.57, [1.32–1.89] × 10^−3^ mm^2^/s) (both *p* < .05). PAs had the lower K^trans^ value (0.12, [0.07–0.18] min^−1^) compared with OBTs (0.28, [0.11–0.50] min^−1^) (*p* < .05). The cutoff values of combined *K*_ep_ and *V*_e_, *D*, and *K*^trans^ to distinguish WTs, MTs, and PAs sequentially were 1.06 min^−1^, 0.28, 1.46 × 10^−3^ mm^2^/s, and 0.21 min^−1^, respectively (accuracy, 89% [71/80], 91% [73/80], 78% [62/80], respectively).

**Conclusion:**

The combined use of DKI and DCE-MRI may help differentiate parotid gland tumors.

**Key Points:**

*• The combined use of DKI and DCE-MRI could facilitate the understanding of the pathophysiological characteristics of parotid gland tumors.*

*• A stepwise diagnostic diagram based on the combined use of DCE-MRI parameters and the diffusion coefficient is helpful for accurate preoperative diagnosis in parotid gland tumors and may further facilitate the clinical management of patients.*

**Supplementary Information:**

The online version contains supplementary material available at 10.1007/s00330-021-08312-y.

## Introduction

Parotid gland tumors (PGTs) contain abundant histological types and subtypes including pleomorphic adenomas (PAs), Warthin tumors (WTs), other benign tumors (OBTs), and malignant tumors (MTs). The treatment strategy and long-term prognosis vary widely depending on the histological type of the tumors [1]. Compared with benign tumors, total parotidectomy with radiotherapy is preferred in the malignancies [2, 3]. In the treatment decision of benign tumors, for patients with PAs, they may require complete surgical excision due to the potential for recurrence and malignant transformation [4], whereas patients with WTs and OBTs, local or superficial parotidectomy, or conservative observation may be sufficient [3]. Therefore, precise preoperative diagnosis is of great importance.

Conventional MRI can delineate lesions concerning the internal structure, morphology, accurate localization, locoregional extension, invasion, and perineural spread of tumors, but the diagnostic performance is limited [3], particularly when benign tumors have similar imaging findings to low-grade malignant tumors showing low signal on T1WI and high signal on T2WI [5]. Advanced MRI techniques like diffusion MRI and perfusion MRI may provide the information of intratumoral water molecular diffusion, microstructural complexity, and capillary blood flows [6, 7]. DKI, a sophisticated modality that quantifies the non-Gaussian behavior of water molecule diffusion, can provide information about heterogeneity and cellularity in vivo with two parameters [8], including diffusion kurtosis (*K*) and diffusion coefficient (*D*), and has been proven to be useful in the field of parotid gland lesions [9, 10]. However, DKI used alone may not fully reflect the pathophysiological characteristics of PGTs. Moreover, DCE-MRI monitors T1 changes in tumor tissues over time after contrast administration of gadolinium to quantify the tumor perfusion and vessel permeability [11, 12]. A quantitative evaluation can be made with perfusion parameters including transfer constant (*K*^trans^), rate constant (*K*_ep_), fractional volume of the extravascular extracellular space (*V*_e_), and initial area under the contrast agent concentration–time curve (iAUC) to identify benign and malignant lesions and monitor tumor responses to treatment in the head and neck [7]. Previous studies showed that quantitative DCE-MRI has played an important part in improving the diagnosis of parotid gland lesions [13–15]. However, those studies may have some limitations, such as the relatively small sample size. Up to now, none of the studies have demonstrated the added value of combining DKI and DCE-MRI for PGT characterization. We hypothesized that the combined use of DKI and DCE-MRI could reflect the discrepancies in diffusion and perfusion of PGTs. Therefore, the purpose of this study was to evaluate whether DKI and DCE-MRI quantitative parameters are beneficial for differentiating PGTs.

## Materials and methods

### Patients

**Fig. 1:**
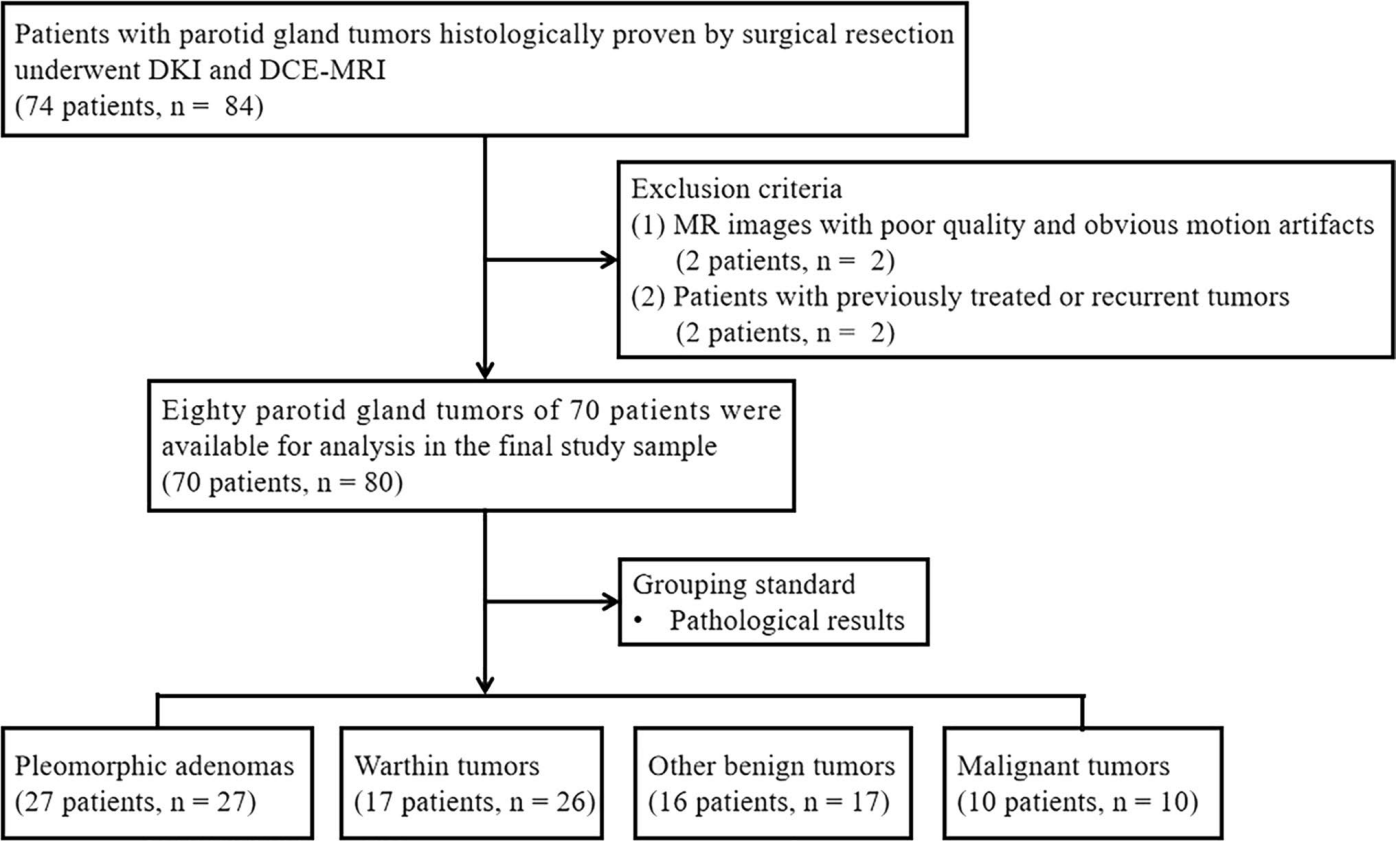
Patients inclusion and exclusion flow diagram

The Institutional Review Board of our hospital approved this retrospective study, and the requirement of written informed consent was waived. Between January 2018 and September 2019, a total of 74 consecutive patients with PGTs histologically proven by surgical resection and available DKI and DCE-MRI sequences were retrospectively recruited. Of these patients, 4 were excluded because of MR images with poor quality and obvious motion artifacts (*n* = 2), and previously treated or recurrent tumors (*n* = 2). Finally, 70 patients with 80 lesions were included in this study and were divided into four groups including PAs (*n* = 27), WTs (*n* = 26), OBTs (*n* = 17), and MTs (*n* = 10) (Fig. 1). The OBTs group included 11 basal cell adenoma (BCA), 3 schwannoma, 1 hemolymphangioma, 1 oncocytic adenoma, and 1 cystadenoma. The MTs group included 4 mucoepidermoid carcinoma, 2 lymphoma, 1 carcinoma ex pleomorphic adenoma, 1 acinic cell carcinoma, 1 salivary duct carcinoma, and 1 mammary analog secretory carcinoma.

### MRI protocol

All patients underwent MRI on a 3.0-T scanner (Skyra; Siemens Healthcare) with a 20-channel head/neck coil. Conventional MRI protocols were obtained first, then DKI using single-shot echo-planar imaging with fat suppression was performed with b-factors of 0, 1000, 1500, 2000, and 2500 s/mm^2^ with diffusion gradient applied along all three orthogonal gradient diffusion directions. T1 mapping was performed prior to DCE-MRI sequence. A dose of 0.1 mmol/kg of gadobenate dimeglumine (Gd-DTPA, MultiHance, Bracco Diagnostics) was intravenously administered through the median cubital vein at the rate of 2 ml/s, followed by a 15-ml flush of saline. The parameters of MR sequences are listed in Supplementary Material 1.

### Image processing and analysis

The DCE-MRI processing was dealt with the commercial software tool (Tissue 4D, Syngo.via; Siemens Healthcare). The post-processing procedures are demonstrated in Supplementary Material 2.

DKI images were processed using prototype software (Body diffusion toolbox, Siemens Healthcare) and Diffusional Kurtosis Estimator (DKE, version 2.5.1, www.musc.edu/cbi). The DKI model is described as the following formula [16]:

$$\frac{S\left(b\right)}{{S}_{0}}=\mathrm{exp}\left(-b\cdot D+\frac{1}{6}{b}^{2}{D}^{2}\cdot K\right),$$

where *D* represents the diffusion coefficient for non-Gaussian distribution and *K* represents the diffusion kurtosis coefficient [17].

For evaluation of DCE-MRI data, the values of quantitative parameters were automatically calculated by placing a single region of interest (ROI) manually in the largest enhanced solid portion of the lesion, excluding blood vessel, hemorrhagic, necrotic, and cystic regions referred to T2WI and contrast-enhanced T1WI. The corresponding ROIs were drawn on DKI maps for *K* and *D* values using the ImageJ software (version 1.8, National Institutes of Health). Image analysis was conducted separately by two radiologists blinded to histological diagnosis (Y.C. and N.H., with 2 and 8 years of experience in head and neck imaging, respectively). The quantitative measurement results of two readers were used to evaluate the interobserver agreement. The quantitative parameter results measured repeatedly by reader 1 with at least 1-month interval were used to evaluate intraobserver agreement. Finally, the measurement results of reader 1 were used for statistical analysis.

### Statistical analysis

All numeric data were tested for normality using Kolmogorov–Smirnov’s test and for variance homogeneity using Levene’s test. The normally distributed variables were expressed as the means ± standard deviation and the non-normally distributed variables were expressed as medians (interquartile ranges, IQRs). A chi-square test was used to compare the discrepancy in sex between benign and malignant tumors. An independent samples *t*-test was made to compare the difference in age between the two groups. Mann–Whitney *U* test was used to compare DCE-MRI and DKI quantitative parameters between benign and malignant PGTs. The Kruskal–Wallis *H* test was made to test overall differences of quantitative parameters among four groups of PGTs. The post hoc test with Bonferroni correction was used for pairwise comparisons when the overall test was statistically significant. The intraclass correlation coefficient (ICC) with 95% confidence intervals (CIs) was used to assess the inter- and intraobserver agreement for quantitative parameters. The ICC > 0.61 was considered good agreement. The receiver operating characteristic (ROC) curve was drawn to ascertain diagnostic performance and optimal cutoff values of quantitative parameters. Then the area under the curve (AUC), sensitivity, specificity, positive predictive value (PPV), negative predictive value (NPV), and accuracy were further calculated. The combination of *K*_ep_ and *V*_e_ values for differentiating WTs from other three entities was based on the logistic regression analysis. The method developed by DeLong et al. [18] was used for comparisons of AUCs. Statistical analysis was performed using MedCalc statistical software (version 15.8, MedCalc), SPSS software (version 26.0, SPSS), and Graphpad Prism (version 6.0, GraphPad Software). *p* < 0.05 was considered statistically significant.

## Results

### Patients characteristics

The main demographic information of patients with PGTs is summarized in Table 1. The statistical results demonstrated that all numeric data were not normally distributed and equal variance except for the age. There was no significant difference in sex (*p* > 0.99) and age distribution (*p* = 0.20) between the benign and malignant groups. Fifty-six patients presented with palpable mass without tenderness, 11 patients felt swelling with pain, and the tumor was found during physical examination in 3 patients. One had facial nerve palsy.

**Table 1:** Patient demographic information

		Benign tumors				
Parameter	Pleomorphic adenomas	Warthin tumors	Other benign tumors	Malignant tumors	Total	*p* value*
No. of patients	27	17	16	10	70	
No. of men	7	14	8	5	34	> .99
Age (year)	45 ± 13 (21–66)	59 ± 9 (42–78)	57 ± 15 (38–78)	46 ± 14 (21–69)	51 ± 14 (21–78)	.20

Mean values are ± standard deviation. Data in parentheses are ranges

^*^p values are for the differences between benign and malignant tumors

### DKI and DCE-MRI analysis between benign and malignant PGTs

The comparisons of quantitative parameters between benign and malignant tumors are summarized in Supplementary Material 3 (Table E2). The *D* value of benign tumors (median 1.50, IQR [1.04–1.86] × 10^−3^ mm^2^/s) was significantly higher than that of malignant tumors (1.10, [0.88–1.29] × 10^−3^ mm^2^/s) (*p* = 0.02). The cutoff value of *D* was 1.24 × 10^−3^ mm^2^/s (AUC, 0.73; accuracy, 69% [55/80]) (Supplementary Material 3, Table E3). Additionally, there were insignificant differences in *K*, *K*^trans^, *K*_ep_, *V*_e_, and iAUC values between the two groups (all *p* > 0.05).

### DKI and DCE-MRI analysis between four groups of PGTs

**Fig. 2:**
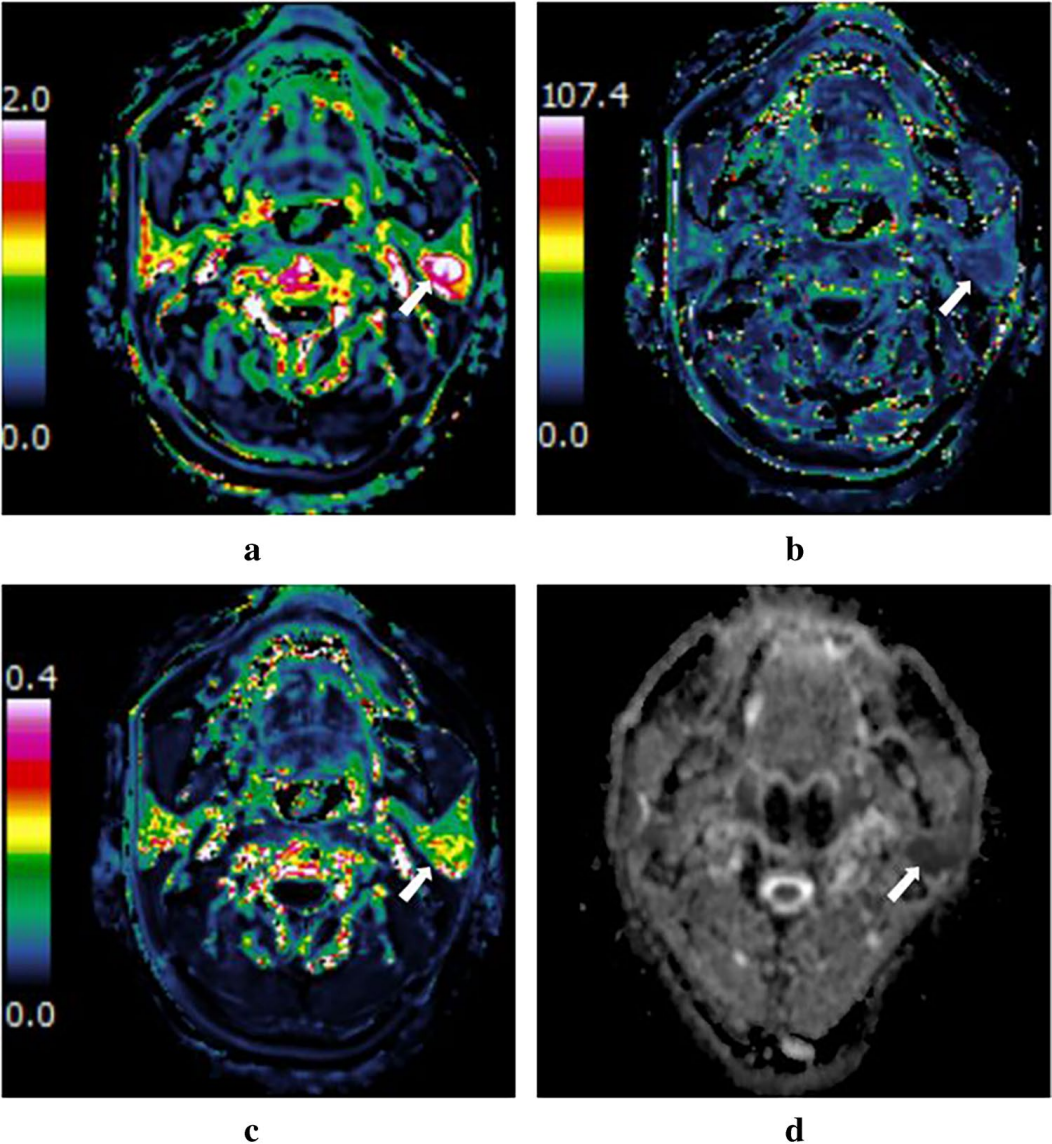
A 67-year-old man with Warthin tumor in the left parotid gland (white arrow). DCE-MRI parameter maps showed the *K*_ep_ (**a**), *V*_e_ (**b**), and *K*^trans^ (**c**) values of 2.02 min^−1^, 0.19, and 0.38 min^−1^, respectively. **d** *D* map demonstrated that the mass showed hypointensity compared with neighboring normal-appearing muscle, with a *D* value of 0.90 × 10^−3^ mm^2^ /s

**Fig. 3:**
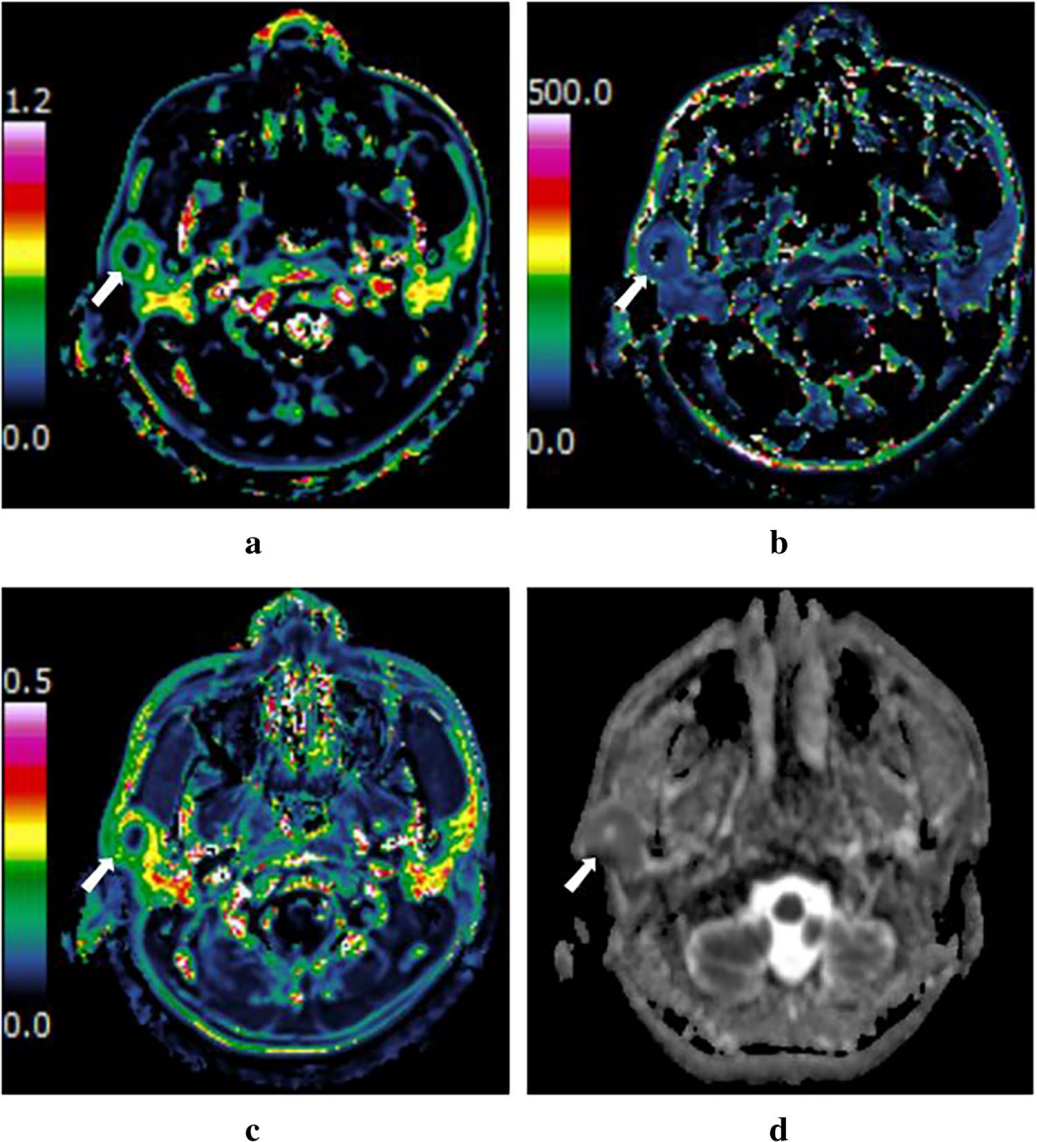
A 24-year-old man with mucoepidermoid carcinoma in the right parotid gland (white arrow). DCE-MRI parameter maps showed the *K*_ep_ (**a**), *V*_e_ (**b**), and *K*^trans^ (**c**) values of 0.90 min^−1^, 0.61, and 0.55 min^−1^, respectively. **d** *D* map demonstrated that the solid portion of the mass showed hypointensity compared with neighboring normal-appearing muscle, with a *D* value of 1.46 × 10^−3^ mm^2^ /s

**Fig. 4:**
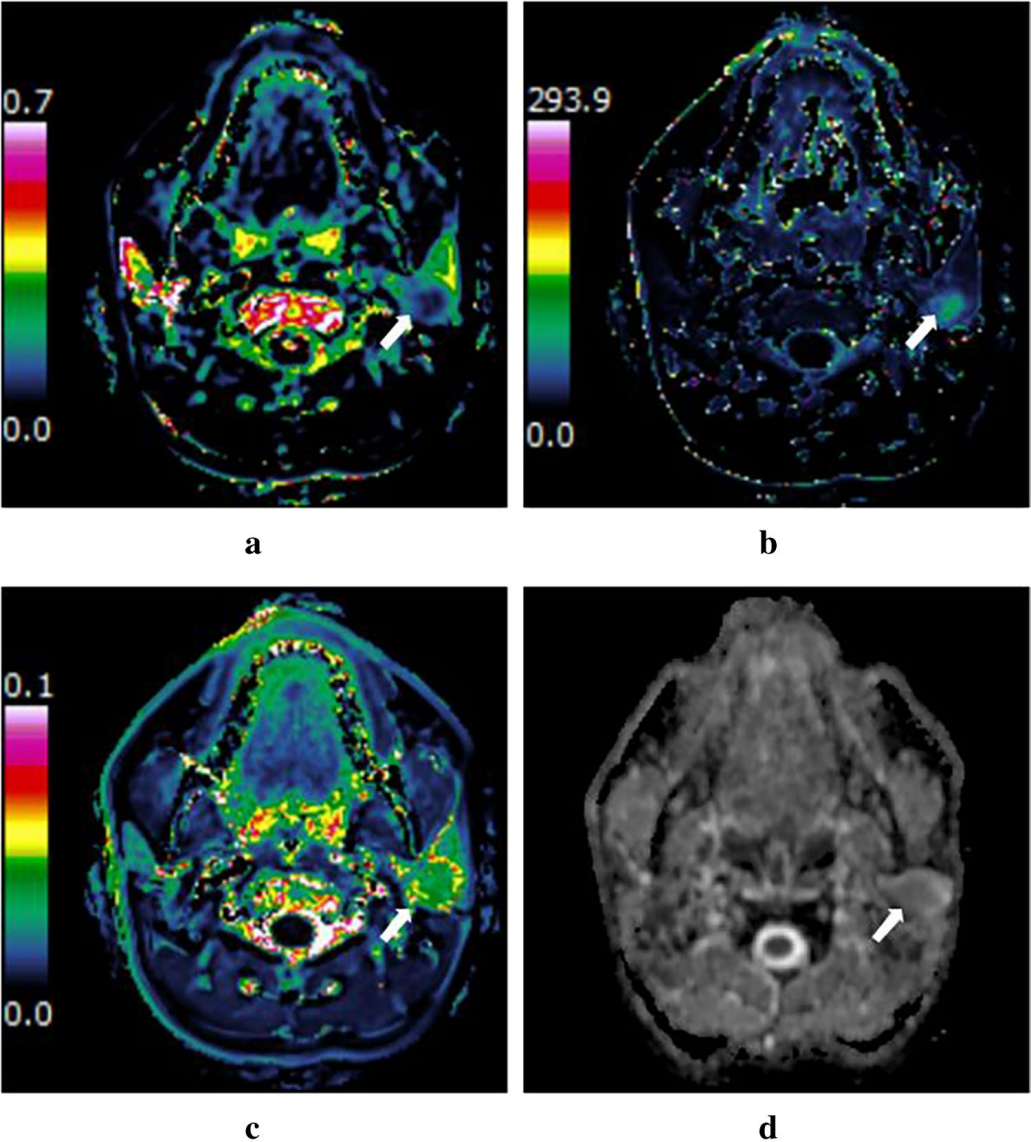
A 34-year-old man with pleomorphic adenoma in the left parotid gland (white arrow). Dynamic contrast-enhanced MRI parameter maps showed the *K*_ep_ (**a**), *V*_e_ (**b**), and *K*^trans^ (**c**) values of 0.30 min^−1^, 0.51, and 0.15 min^−1^, respectively. **d** *D* map demonstrated that the mass showed slightly hypointensity compared with neighboring normal-appearing muscle, with a *D* value of 1.85 × 10^−3^ mm^2^ /s

**Fig. 5:**
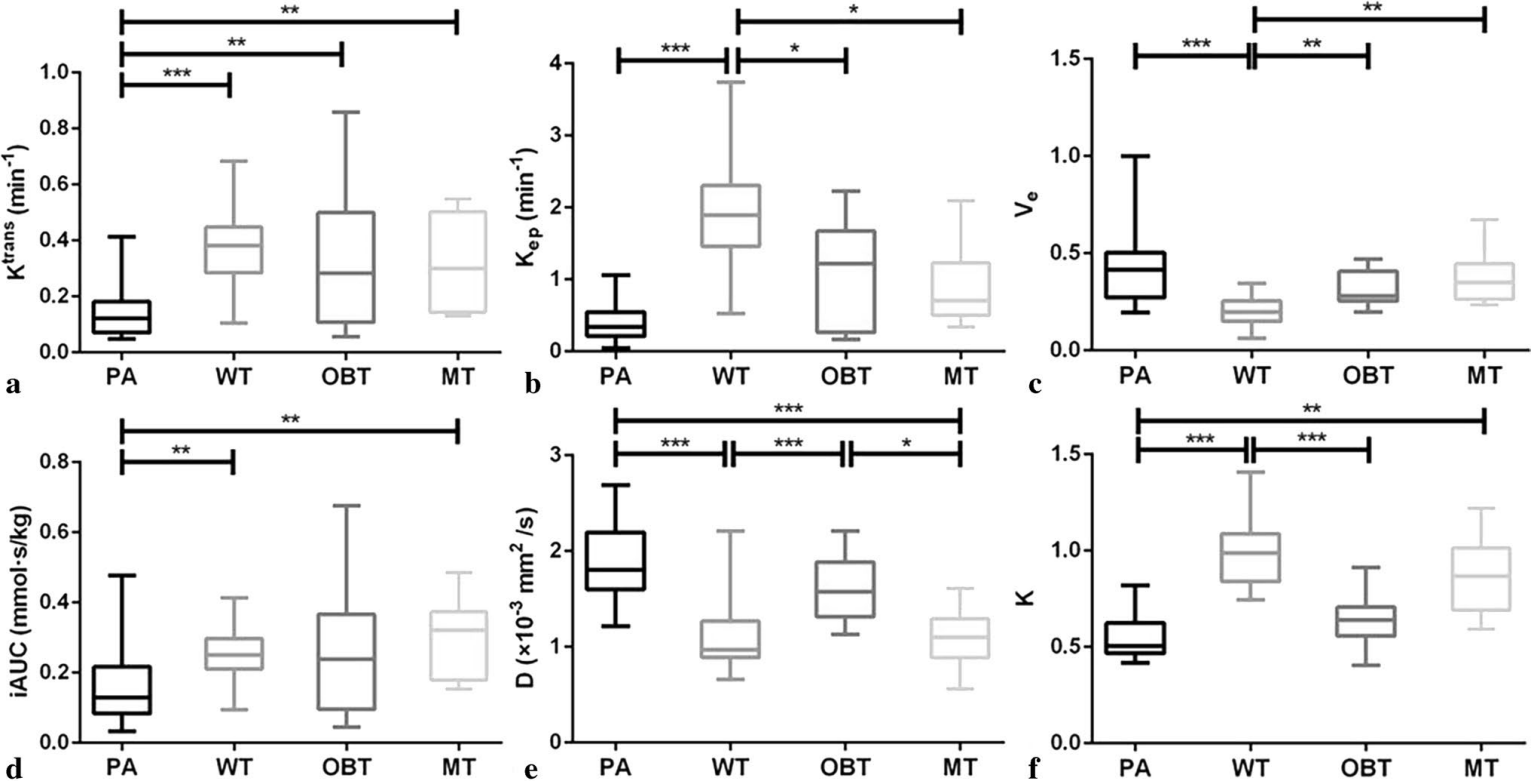
Box-and-whisker plots show mean DCE-MRI and DKI quantitative parameters for all cases. Boundaries of boxes indicate 25th and 75th percentiles, and lines in boxes indicate medians. *PA* pleomorphic adenoma, *WT* Warthin tumor, *OBT* other benign tumor, *MT* malignant tumor. *p < .05; **p < .01; ***p < .001

The Kruskal–Wallis *H* test revealed that there were statistically significant differences in all quantitative parameters among different groups of PGTs (all *p* < 0.001), and the comparisons of quantitative parameters of PGTs are summarized in Table 2. For DCE-MRI parameters, as exhibited in Figs. 2, 3, 4, and 5, the *K*^trans^ value of PAs (0.12, [0.07–0.18] min^−1^) was significantly lower than that of WTs (0.38, [0.28–0.45] min^−1^), OBTs (0.28, [0.11–0.50] min^−1^), and MTs (0.30, [0.14–0.50] min^−1^) (adjusted *p* < 0.001, = 0.008, and 0.009, respectively). Moreover, the *K*_ep_ value of WTs (1.89, [1.46–2.31] min^−1^) was significantly higher than that of PAs (0.34, [0.21–0.55] min^−1^), OBTs (1.22, [0.27–1.67] min^−1^), and MTs (0.71, [0.50–1.23] min^−1^) (adjusted *p* < 0.001, = 0.01, and 0.047, respectively). Meanwhile, the *V*_e_ value of WTs (0.20, [0.15–0.25]) was significantly lower than that of PAs (0.36, [0.24–0.43]), OBTs (0.28, [0.25–0.41]), and MTs (0.35, [0.26–0.45]) (adjusted *p* < 0.001, = 0.001, and 0.001, respectively). Additionally, the iAUC value of PAs (0.13, [0.08–0.22] mmol·s/kg) was significantly lower than that of WTs (0.25, [0.21–0.30] mmol·s/kg) and MTs (0.32, [0.18–0.37] mmol·s/kg) (adjusted *p* = 0.002 and 0.007, respectively).

**Table 2:** DKI and DCE-MRI parameters of PGTs and Kruskal–Wallis *H* test with Bonferroni correction

					*p* value^a^			*p* value^b^			
Parameters	PA	WT	OBT	MT		PA vs WT	PA vs OBT	PA vs MT	WT vs OBT	WT vs MT	OBT vs MT
DKI parameters											
* K*	0.51 (0.47–0.62)	0.99 (0.84–1.09)	0.64 (0.56–0.71)	0.87 (0.69–1.01)	< .001*	< .001*	.06	.001*	< .001*	> .99	.12
* D* (× 10^−3^ mm^2^/s)	1.81 (1.60–2.20)	0.97 (0.89–1.27)	1.57 (1.32–1.89)	1.10 (0.88–1.29)	< .001*	< .001*	.90	< .001*	< .001*	> .99	.03*
DCE-MRI parameters											
*K*^trans^ (min^−1^)	0.12 (0.07–0.18)	0.38 (0.28–0.45)	0.28 (0.11–0.50)	0.30 (0.14–0.50)	< .001*	< .001*	.008*	.009*	.83	> .99	> .99
*K*_ep_ (min^−1^)	0.34 (0.21–0.55)	1.89 (1.46–2.31)	1.22 (0.27–1.67)	0.71 (0.50–1.23)	< .001*	< .001*	.06	.20	.01*	.047*	> .99
*V*_e_	0.36 (0.24–0.43)	0.20 (0.15–0.25)	0.28 (0.25–0.41)	0.35 (0.26–0.45)	< .001*	< .001*	> .99	> .99	.001*	.001*	> .99
iAUC (mmol·s/kg)	0.13 (0.08–0.22)	0.25 (0.21–0.30)	0.24 (0.10–0.37)	0.32 (0.18–0.37)	< .001*	.002*	.09	.007*	> .99	> .99	> .99

*DKI* diffusion kurtosis imaging, *DCE-MRI* dynamic contrast-enhanced MRI, *PGTs* parotid gland tumors, *PA* pleomorphic adenoma, *WT* Warthin tumor, *OBT* other benign tumor, *MT* malignant tumor, *K* diffusion kurtosis, *D* diffusion coefficient, *K*^*trans*^ transfer constant from plasma to extravascular extracellular space, *K*_*ep*_ rate constant from extravascular extracellular space to plasma, *V*_*e*_ fractional volume of the extravascular extracellular space, *iAUC* initial area under the contrast agent concentration–time curve. Data are medians with interquartile ranges in parentheses. *p* values^a^ from Kruskal–Wallis *H* test. *p* values^b^ are adjusted for pairwise comparison by using post hoc test with Bonferroni correction

^*^Significant difference (*p* < .05)

For DKI parameters, the *D* values of both MTs (1.10, [0.88–1.29] × 10^−3^ mm^2^/s) and WTs (0.97, [0.89–1.27] × 10^−3^ mm^2^/s) were significantly lower than those of PAs (1.81, [1.60–2.20] × 10^−3^ mm^2^/s) (both adjusted *p* < 0.001 for MTs and WTs) and OBTs (1.57, [1.32–1.89] × 10^−3^ mm^2^/s) (adjusted *p* = 0.03 for MTs and < 0.001 for WTs), respectively. Moreover, the *K* value of PAs (0.51, [0.47–0.62]) was significantly lower than that of MTs (0.87, [0.69–1.01]) (adjusted *p* = 0.001); the *K* value of WTs (0.99, [0.84–1.09]) was higher than that of PAs and OBTs (0.64, [0.56–0.71]) (both adjusted *p* < 0.001).

The diagnostic performances between the four groups are summarized in Table 3. ROC analyses showed that in differentiating PAs from WTs, *K* (cutoff value, ≤ 0.72; AUC, 0.99; accuracy, 98% [52/53]) showed optimal diagnostic performance, which was better than that of *V*_e_ and iAUC (both *p* = 0.003), but the difference in AUC between *K* and *K*^trans^, *K*_ep_, or *D* did not reach significance (*p* = 0.08, 0.50, and 0.20, respectively). In differentiating PAs from OBTs, the cutoff value of *K*^trans^ was 0.21 min^−1^ (AUC, 0.77; accuracy, 80% [35/44]). In differentiating PAs from MTs, *D* (cutoff value, > 1.46 × 10^−3^ mm^2^/s; AUC, 0.96; accuracy, 92% [34/37]) showed optimal diagnostic performance, but the difference in AUC between *D* and *K*^trans^, iAUC, or K did not reach significance (*p* = 0.14, 0.08, and 0.40, respectively). In differentiating WTs from OBTs, *K* (cutoff value, > 0.72; AUC, 0.97; accuracy, 93% [40/43]) showed optimal diagnostic performance, which was significantly better than that of *K*_ep_ (*p* = 0.01), but the difference in AUC between *K* and *V*_e_ or *D* did not reach significance (*p* = 0.13 and 0.11, respectively). In differentiating WTs from MTs, *V*_e_ (cutoff value, > 0.23; AUC, 0.92; accuracy, 78% [28/36]) showed a higher AUC than *K*_ep_, but the difference did not reach significance (*p* = 0.33). The cutoff value of *D* was 1.24 × 10^−3^ mm^2^/s in differentiating OBTs from MTs (AUC, 0.88; accuracy, 82% [22/27]).

**Table 3:** Optimal cutoff values and diagnostic performance of DKI and DCE-MRI parameters for differentiating four groups of PGTs

Parameters	Cutoff value	AUC	Sensitivity (%)	Specificity (%)	Accuracy (%)	PPV (%)	NPV (%)
Pleomorphic adenomas vs. Warthin’s tumors							
*K*^trans^	0.21	0.93	93 (25/27)	88 (23/26)	91 (48/53)	89 (25/28)	92 (23/25)
*K*_ep_	1.06	0.99	100 (27/27)	92 (24/26)	96 (51/53)	93 (27/29)	100 (24/24)
*V*_e_	0.28	0.82	63 (17/27)	96 (25/26)	79 (42/53)	94 (17/18)	71 (25/35)
iAUC	0.19	0.83	74 (20/27)	88 (23/26)	81 (43/53)	87 (20/23)	77 (23/30)
*D*	1.43	0.95	93 (25/27)	96 (25/26)	94 (50/53)	96 (25/26)	93 (25/27)
*K*	0.72	0.99	96 (26/27)	100 (26/26)	98 (52/53)	100 (26/26)	96 (26/27)
Pleomorphic adenomas vs. other benign tumors							
*K*^trans^	0.21	0.77	93 (25/27)	59 (10/17)	80 (35/44)	78 (25/32)	83 (10/12)
Pleomorphic adenomas vs. malignant tumors							
*K*^trans^	0.13	0.86	59 (16/27)	100 (10/10)	70 (17/37)	100 (16/16)	48 (10/21)
iAUC	0.15	0.84	59 (16/27)	100 (10/10)	70 (17/37)	100 (16/16)	48 (10/21)
*D*	1.46	0.96	93 (25/27)	90 (9/10)	92 (34/37)	96 (25/26)	82 (9/11)
*K*	0.54	0.93	70 (19/27)	100 (10/10)	78 (29/37)	100 (19/19)	56 (10/18)
Warthin’s tumors vs. other benign tumors							
*K*_ep_	1.51	0.79	73 (19/26)	76 (13/17)	74 (32/43)	83 (19/23)	65 (13/20)
*V*_e_	0.23	0.88	69 (18/26)	94 (16/17)	79 (34/43)	95 (18/19)	67 (16/24)
*D*	1.05	0.90	73 (19/26)	100 (17/17)	84 (36/43)	100 (19/19)	71 (17/24)
*K*	0.72	0.97	100 (26/26)	82 (14/17)	93 (40/43)	90 (26/29)	100 (14/14)
Warthin’s tumors vs. malignant tumors							
*K*_ep_	0.98	0.86	92 (24/26)	80 (8/10)	89 (32/36)	92 (24/26)	80 (8/10)
*V*_e_	0.23	0.92	69 (18/26)	100 (10/10)	78 (28/36)	100 (18/18)	56 (10/18)
Other benign tumors vs. malignant tumors							
*D*	1.24	0.88	82 (14/17)	80 (8/10)	82 (22/27)	88 (14/16)	73 (8/11)

*DKI* diffusion kurtosis imaging, *DCE-MRI* dynamic contrast-enhanced MRI, *PGTs* parotid gland tumors, *AUC* the area under the curve, *PPV* positive predictive value, *NPV* negative predictive value, *K* diffusion kurtosis, *D* diffusion coefficient, *K*^*trans*^ transfer constant from plasma to extravascular extracellular space, *K*_*ep*_ rate constant from extravascular extracellular space to plasma, *V*_*e*_ fractional volume of the extravascular extracellular space, *iAUC* initial area under the contrast agent concentration–time curve. Data in parentheses are the numerator and denominator used to calculate percentages. *K*_ep_ and *K*^trans^ values are expressed in min^−1^; iAUC values are expressed in mmol·s/kg. *D* values are expressed in × 10^−3^ mm^2^/s

Excellent inter- and intraobserver agreement for DKI and DCE-MRI parameters was achieved with ICCs ranging from 0.92 to 0.99 (see Supplementary Material 4 for details).

### DKI and DCE-MRI analysis between solid and predominantly cystic of WTs

To overcome the possible influence in DKI and DCE-MRI parameters caused by heterogeneity of Warthin tumors, we further compared these parameters derived from the solid portion between Warthin tumors with solid and predominantly cystic forms. Of 26 Warthin tumors, 20 (77%) were presented as predominantly solid masses, which included completely solid or solid tumor with small cystic components, and 6 (23%) were presented as predominantly cystic entities. No significant differences were found in all parameters between predominantly solid and predominantly cystic Warthin tumors (*p* = 0.12, 0.05, 0.70, 0.98, 0.66, and 0.22 for *K*^trans^, *K*_ep_, *V*_e_, iAUC, *D*, and *K* value, respectively).

### Stepwise discrimination among four groups of PGTs using DKI and DCE-MRI

Initially, PAs, OBTs, and MTs were classified as one group for the reason that there were significant differences in *K*_ep_ and *V*_e_ values between these three groups of tumors and WTs. Both *K*_ep_ and *V*_e_ values helped discriminate WTs from PAs, OBTs, and MTs, with a cutoff value of > 1.06 min^−1^ for *K*_ep_ (accuracy, 84% [67/80]) and ≤ 0.28 for *V*_e_ (accuracy, 74% [59/80]). Moreover, the combination of *K*_ep_ and *V*_e_ values generated the better diagnostic performance (accuracy, 89% [71/80]) than *V*_e_ (AUC, 0.93 vs 0.86; *p* = 0.03) and was finally applied to distinguish WTs from the other three groups of PGTs (Table 4).

**Table 4:** Optimal cutoff values and diagnostic performance of DKI and DCE-MRI parameters for stepwise discrimination

Parameters	Cutoff value	AUC	Sensitivity (%)	Specificity (%)	Accuracy (%)	PPV (%)	NPV (%)
WTs vs. PAs + OBTs + MTs							
*K*_ep_	1.06	0.90	92 (24/26)	80 (43/54)	84 (67/80)	69 (24/35)	96 (43/45)
*V*_e_	0.28	0.86	96 (25/26)	63 (34/54)	74 (59/80)	56 (25/45)	97 (34/35)
*K*_ep +_ *V*_e_	–	0.93	92 (24/26)	87 (47/54)	89 (71/80)	77 (24/31)	96 (47/49)
MTs vs. PAs + OBTs							
*D*	1.46	0.93	90 (9/10)	84 (37/44)	85 (46/54)	56 (9/16)	97 (37/38)
PAs vs. OBTs							
*K*^trans^	0.21	0.77	93 (25/27)	59 (10/17)	80 (35/44)	78 (25/32)	83 (10/12)

*DKI* diffusion kurtosis imaging, *DCE-MRI* dynamic contrast-enhanced MRI, *AUC* area under the ROC curve, *PPV* positive predictive value, *NPV* negative predictive value, *WTs* Warthin tumors, *PAs* pleomorphic adenomas, *OBTs* other benign tumors, *MTs* malignant tumors, *K*_*ep*_ rate constant from extravascular extracellular space to plasma, *V*_*e*_ fractional volume of the extravascular extracellular space, *D* diffusion coefficient, *K*^*trans*^ transfer constant from plasma to extravascular extracellular space. Data in parentheses are the numerator and denominator used to calculate percentages. *K*_ep_ and *K*^trans^ values are expressed in min^−1^; *D* values are expressed in × 10^−3^ mm^2^/s

Subsequently, PAs and OBTs were classified as one group for the reason that lower *D* value was found in MTs than the other two entities. The *D* value was useful for differentiating MTs from PAs and OBTs, with a cutoff value of ≤ 1.46 × 10^−3^ mm^2^/s (accuracy, 85% [46/54]) (Table 4).

**Fig. 6:**
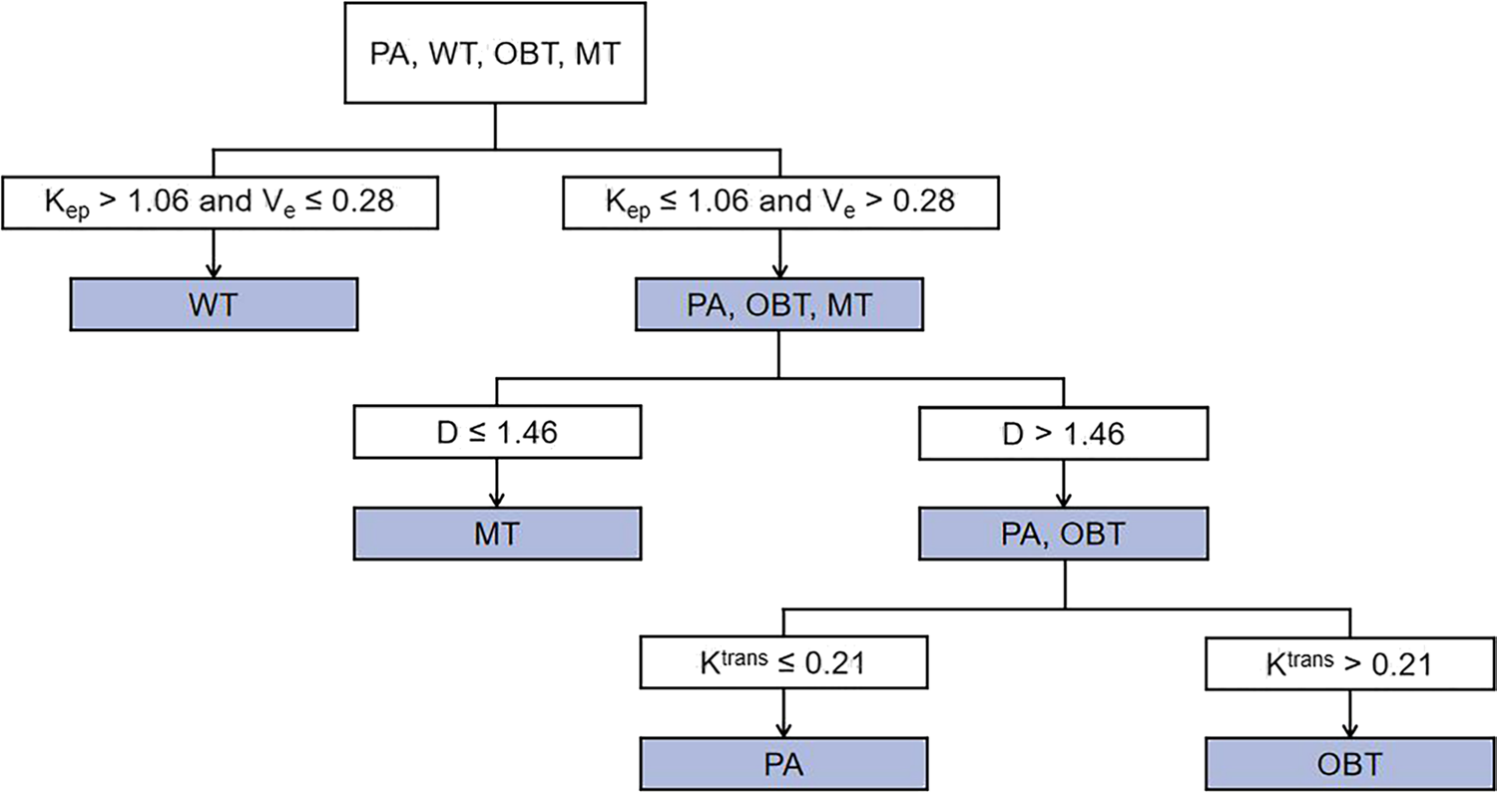
Graph shows stepwise discrimination of four groups of parotid gland tumors, including Warthin tumors, malignant tumors, pleomorphic adenomas, and other benign tumors using DCE-MRI parameters and diffusion coefficient. *PA* pleomorphic adenoma, *WT* Warthin tumor, *OBT* other benign tumor, *MT* malignant tumor. Units for quantitative parameters: *K*_ep_ and *K*^trans^ are expressed in min^−1^; D are expressed in × 10^−3^ mm^2^ /s

Eventually, the *K*^trans^ value was used to differentiating PAs from OBTs resulted from the lower *K*^trans^ value of PAs with a cut-off value of ≤ 0.21 min^−1^ (accuracy, 80% [35/44]) (Table 4). From this aspect, a stepwise distinguishable diagnostic diagram was put up for discriminating the four groups of PGTs consisting of PAs, WTs, OBTs, and MTs (Table 5 and Fig. 6). The diagnostic diagram provided high accuracy for the differential diagnosis of WTs and MTs of 89% (71/80) and 91% (73/80), respectively, and modest accuracy for both PAs and OBTs of 78% (62/80).

**Table 5:** Diagnostic accuracy with the combination of DKI and DCE-MRI parameters for the differentiation of PGTs

		DKI/DCE-MRI criteria		
Parameters	*K*_ep_ + *V*_e_	*D*	*K*^trans^	Diagnostic accuracy (%)
WT	*K*_ep_ > 1.06, *V*_e_ ≤ 0.28	–	–	89 (71/80)
MT	*K*_ep_ ≤ 1.06, *V*_e_ > 0.28	≤ 1.46	–	91 (73/80)
PA	*K*_ep_ ≤ 1.06, *V*_e_ > 0.28	> 1.46	≤ 0.21	78 (62/80)
OBT	*K*_ep_ ≤ 1.06, *V*_e_ > 0.28	> 1.46	> 0.21	78 (62/80)

*DKI* diffusion kurtosis imaging, *DCE-MRI* dynamic contrast-enhanced MRI, *PGTs* parotid gland tumors, *WT* Warthin tumor, *MT* malignant tumor, *PA* pleomorphic adenoma, *OBT* other benign tumor, *K*_*ep*_ rate constant from extravascular extracellular space to plasma, *V*_*e*_ fractional volume of the extravascular extracellular space, *D* diffusion coefficient, *K*^*trans*^ transfer constant from plasma to extravascular extracellular space. Data in parentheses are the numerator and denominator used to calculate percentages. *K*_ep_ and *K*^trans^ values are expressed in min^−1^; *D* values are expressed in × 10^−3^ mm^2^/s

## Discussion

DKI and DCE-MRI have been widely used in head and neck regions [19, 20]. However, to our knowledge, the diagnostic performance of combined DKI and DCE-MRI models in the field of PGTs has not been assessed in the existing studies. Our current study showed that DKI and DCE-MRI can elucidate the diffusion and perfusion characteristics of PGTs and provided a stepwise diagnostic diagram to distinguish common PGTs with modest to high accuracy (78–91%). These findings suggest that DKI and DCE-MRI quantitative parameters may facilitate the understanding of the pathophysiological characteristics of PGTs.

The *D* value derived from DKI is the corrected diffusion-related coefficient for non-Gaussian bias, and there is a strong inverse association with the tumor cellular density and nucleus-to-cytoplasm ratios [19]. Previously, Qian et al. [10] showed that no significant difference was found in the *D* value between the benign and the malignant tumors. They implied that low *D* value in WTs mainly accounted for low *D* value in benign PGTs and there was possibly a slight difference of *D* value by increasing the sample size. In this study, with a relatively big sample size, the mean *D* value was the only significant parameter in distinguishing the benignity from the malignancy, which was consistent with the hypothesis. In subgroup comparisons, a previous study [9] demonstrated that PAs had higher *D* value whereas WTs and MTs had relatively lower *D* value, which was in good agreement with our results. Histologically, the high *D* value in PAs resulted from the abundant myxoid and chondroid matrices [10]. Flourishing cells, enlarged nuclei, and smaller extracellular space in MTs [21] and high cellularity that resulted from rich lymphoid tissue–related interstitium in WTs [22, 23] help explain the low *D* value. Notably, in this study, *D* was used for the second step in the stepwise diagnostic diagram instead of *K*, due to the fact that the difference of *K* value between OBTs and MTs was not statistically significant, which was incompatible with the previous study [9]. The chief reasons for it may be different histopathological subtypes of OBTs and MTs and different *b*-value selection in DKI scan parameters in the two studies [24]. However, ROC curve analysis showed that the *K* value can provide a high accuracy in differentiating WTs from PAs and OBTs. The *K* value can quantify the degree of non-Gaussian distribution and tend to move together with cellular heterogeneity and tissue complexity [21]. The higher *K* value of WTs might be attributed to the complex microstructure within tumor, including various proportions of epithelia with papillary proliferation, lymphoid tissue, and cystic components filled with mucoid in histopathology [25]. Hence, our findings suggest that the *K* value evaluated preoperatively may aid in differentiating PGTs.

*K*^trans^ derived from DCE-MRI chiefly depends on blood flow in tissue and capillary permeability [19, 26]. Our findings discovered that *K*^trans^ was the only useful imaging marker for distinguishing PAs from OBTs, which was inconsistent with the previous study [14]. In our study, BCAs accounted for the majority of OBTs, which possibly to some extent affected the *K*^trans^ value in the OBTs. Hence, the significant differences in *K*^trans^ value were related to the comparative higher *K*^trans^ value of BCAs. Histologically, compared with PAs, BCAs are short of mesenchymal component and chondromyxoid stroma but have a great deal of vascular architecture [27]. The different histological features may be the dominant reason; another one may be various pathological types but small sample size in OBTs in our study. Further studies with a bigger sample size are mandatory to analyze the differences of quantitative parameters between PAs and OBTs. *V*_e_ has a strong correlation with tissue necrosis and cellularity and *K*_ep_ is equal to the ratio of *K*^trans^ to *V*_e_ [19, 26]. In our study, WTs had the highest *K*_ep_ value but lowest *V*_e_ value than the other three groups, which was in accord with the previous study [13, 14]. In the study by Yabuuchi et al. [13], the differences in *K*_ep_ and *V*_e_ values reached statistical significance among four types of PGTs and particularly, the use of the *K*_ep_ value extremely promoted the diagnostic efficacy in the decision tree analysis. Histologically, limited extracellular and extravascular space in WTs [14] results in high *K*_ep_ value but low *V*_e_ value. Accordingly, with the characteristic presentations of perfusion parameters (high *K*_ep_ value and low *V*_e_ value), WTs could be effectively differentiated from the other three groups.

In our current study, the improved diagnostic performance was found in the combination of *K*_ep_ and *V*_e_ values for differentiating WTs from the other three groups, indicating *K*_ep_ and *V*_e_ values could be the optimal quantitative parameters for the diagnosis of WTs. Moreover, favorable sensitivity, specificity, and accuracy were also investigated in *D* value for the differentiation of MTs from PAs and OBTs, implying *D* value may conduce to the differential diagnosis. Furthermore, our study showed that *K*^trans^ value avails to further discriminating PAs from OBTs. As a result, in the manner of the stepwise diagram combining DCE-MRI- and DKI-derived parameters, these common histological types of PGTs could be well differentiated. Regardless, the validation and correction of the criteria still need to be made with a larger sample size in further studies.

There are several limitations to our study. First, this was a retrospective study that may generate bias in case selection and the sample size of patients with malignant tumor was relatively small. Further study with a larger sample size is required to confirm our results. Second, magnetic susceptibility artifacts with anatomic distortions may inevitably exert an influence on the measurements and corresponding results. Various available DKI sequences like the readout-segmented echo-planar imaging (RS-EPI) technique may be offered as a solution. Third, our study applied DKI and DCE-MRI for PGTs. Further studies with multimodal imaging using intravoxel incoherent motion (IVIM) and arterial spin labeling (ASL) will improve the results.

In conclusion, our study demonstrated that DKI and DCE-MRI are useful for characterizing common PGTs. Additionally, a stepwise diagnostic diagram was put up based on the combined use of DCE-MRI parameters and the diffusion coefficient to improve the diagnostic ability. In the future, studies with a more abundant and larger sample size are necessary to help scrutinize, optimize, and validate our stepwise diagram and expand the applications of DKI and DCE-MRI to other parotid gland diseases.

## Supplementary Information

Below is the link to the electronic supplementary material.

Supplementary file1 (DOCX 38 KB)

## Abbreviations

BCA: Basal cell adenoma
*D*: Diffusion coefficient
DCE-MRI: Dynamic contrast-enhanced MRI
DKI: Diffusion kurtosis imaging
iAUC: Initial area under the contrast agent concentration–time curve
IQR: Interquartile range
*K*: Diffusion kurtosis
*K*_ep_: Rate constant from extravascular extracellular space to plasma
*K*^trans^: Transfer constant from plasma to extravascular extracellular space
MT: Malignant tumor
OBT: Other benign tumor
PA: Pleomorphic adenoma
PGT: Parotid gland tumor
*V*_e_: Fractional volume of the extravascular extracellular space
WT: Warthin tumor

## Declarations

### Guarantor

The scientific guarantor of this publication is Prof. Dairong Cao, MD, The First Affiliated Hospital of Fujian Medical University.

### Statistics and biometry

No complex statistical methods were necessary for this paper.

### Study subjects and cohort overlap

Some patients in the present study cohort overlapped one of our studies, in which we used readout segmentation of long variable echo-trains diffusion-weighted imaging and quantitative dynamic contrast-enhanced MRI (DCE-MRI) with ROI measurements to differentiate parotid tumors. As for the DCE-MRI part, 69 cases (56.1%) overlapped in these two studies. In that study, the temporal resolution, duration of dynamic sequence and diffusion technique, and calculated mode were far different from our present study.

### Methodology

• Retrospective

• diagnostic or prognostic study

• performed at one institution
