# Supplementary material for: Diffusion kurtosis imaging and dynamic contrast-enhanced MRI for the differentiation of parotid gland tumors
Source: Eur Radiol. 2021 Oct 12;32(4):2748–59. doi: 10.1007/s00330-021-08312-y (PMC8921043; doi:10.1007/s00330-021-08312-y)
Supplement: Supplementary file 1 — Supplementary file1 (DOCX 38 KB) [file 330_2021_8312_MOESM1_ESM.docx]

**Supplementary Materials**

**Supplementary Material 1**

**Table E1.** The Parameters of MR Sequences

| Sequence | TR (msec) | TE (msec) | FOV (mm) | Matrix | Slice Thickness (mm) | FA (°) | Average | Acquisition Time |
| --- | --- | --- | --- | --- | --- | --- | --- | --- |
| Conventional MRI |  |  |  |  |  |  |  |  |
| Axial T2WI | 3690 | 83 | 220 × 220 | 320 × 320 | 4 | 100 | 2 | 1 min 25 sec |
| Axial T1WI | 739 | 9.9 | 220 × 206 | 320 × 240 | 4 | 128 | 2 | 2 min 47 sec |
| CE-T1WI | 739 | 9.9 | 220 × 206 | 320 × 240 | 4 | 128 | 1 | 1 min 30 sec |
| DKI | 5100 | 83 | 220 × 220 | 150 × 135 | 4 |  | 2 (b = 0)  3 (b = 1000)  4 (b = 1500)  5 (b = 2000)  5 (b = 2500) | 4 min 46 sec |
| DCE-MRI |  |  |  |  |  |  |  |  |
| T1 mapping | 4.95 | 1.75 | 240 × 240 | 192 × 154 | 2 | 2 and 15 | 1 | 1 min 47 sec |
| Dynamic CE-T1WI* | 5.08 | 1.79 | 240 × 217 | 192 × 154 | 3.5 | 15 | 1 | 5 min |

*TR* repetition time, *TE* echo time, *FOV* field of view, *FA* flip angle, *T2WI* T2-weighted imaging, *T1WI* T1-weighted imaging, *CE-T1WI* contrast-enhanced T1-weighted imaging, *DKI* diffusion kurtosis imaging, *DCE-MRI* dynamic contrast-enhanced MRI.

* Temporal resolution, 6 s/dynamic; number of dynamics, 50.

**Supplementary Material 2**

The DCE-MRI processing was dealt with the commercial software tool (Tissue 4D, Syngo.via, Siemens Healthcare). The post-processing procedure included motion correction, alignment, and processing.

1. Motion correction of dynamic series;
2. Alignment of pre-contrast and morphology to dynamic reference: Measured T1 and T2-weighted images or T1-weighted dynamic contrast-enhanced MR images were selected, respectively;
3. Processing step:
4. A volume of interest (VOI) was drawn on DCE-MRI images, which contained the lesion and adjacent normal tissue in the parotid gland;
5. The concentration curve of the VOI was generated according to the Tofts model [1];
6. A population-based arterial input function (AIF) was used and set to “fast” [2], “intermediate” [3], or “slow” [4] model with the minimum of chi-square parameter; the chi-square parameter is an error measure of the fit. The smaller the parameter, the better the fit;
7. Computation of parametric maps (K^trans^, K_ep_, V_e_, and iAUC) of the VOI according to the Tofts model.

**Supplementary Material 3**

**Table E2.** DKI and DCE-MRI Parameters of Benign and Malignant PGTs and Mann-Whitney U Test

| Parameters | Benign Tumors | Malignant Tumors | *p* Value |
| --- | --- | --- | --- |
| DKI parameters |  |  |  |
| K | 0.67 (0.51-0.92) | 0.87 (0.69-1.01) | .07 |
| D (×10^-3^ mm^2^/s) | 1.50 (1.04-1.86) | 1.10 (0.88-1.29) | .02* |
| DCE-MRI parameters |  |  |  |
| K^trans^ (min^-1^) | 0.22 (0.11-0.40) | 0.30 (0.14-0.50) | .24 |
| K_ep_ (min^-1^) | 0.99 (0.32-1.84) | 0.71 (0.50-1.23) | .84 |
| V_e_ | 0.26 (0.20-0.37) | 0.35 (0.26-0.45) | .07 |
| iAUC (mmol·s/kg) | 0.21 (0.12-0.29) | 0.32 (0.18-0.37) | .05 |

*DKI* diffusion kurtosis imaging, *DCE-MRI* dynamic contrast-enhanced MRI, *PGTs* parotid gland tumors, *K* diffusion kurtosis, *D* diffusion coefficient, *K^trans^* transfer constant from plasma to extravascular extracellular space, *K_ep_* rate constant from extravascular extracellular space to plasma, *V_e_* fractional volume of the extravascular extracellular space, *iAUC* initial area under the contrast agent concentration-time curve. Data are medians with interquartile ranges in parentheses. *p* values from Mann-Whitney U test.

* Significant difference (*p* < .05).

**Table E3.** Optimal Cut-off Values and Diagnostic Performance of DKI and DCE-MRI Parameters for Differentiating Benign and Malignant PGTs

| Parameters | Cut-off Value | AUC | Sensitivity (%) | Specificity (%) | Accuracy (%) | PPV (%) | NPV (%) |
| --- | --- | --- | --- | --- | --- | --- | --- |
| DKI parameters |  |  |  |  |  |  |  |
| K | 0.82 | 0.68 | 66 (46/70) | 70 (7/10) | 66 (53/80) | 94 (46/49) | 23 (7/31) |
| D | 1.24 | 0.73 | 67 (47/70) | 80 (8/10) | 69 (55/80) | 96 (47/49) | 26 (8/31) |
| DCE-MRI parameters |  |  |  |  |  |  |  |
| K^trans^ | 0.13 | 0.62 | 33 (23/70) | 100 (10/10) | 41 (33/80) | 100 (23/23) | 18 (10/57) |
| K_ep_ | 0.98 | 0.52 | 50 (35/70) | 20 (2/10) | 46 (37/80) | 81 (35/43) | 5 (2/37) |
| V_e_ | 0.23 | 0.68 | 36 (25/70) | 100 (10/10) | 44 (35/80) | 100 (25/25) | 18 (10/55) |
| iAUC | 0.30 | 0.69 | 79 (55/70) | 60 (6/10) | 76 (61/80) | 93 (55/59) | 29 (6/21) |

*DKI* diffusion kurtosis imaging, *DCE-MRI* dynamic contrast-enhanced MRI, *PGTs* parotid gland tumors, *AUC* the area under the curve, *PPV* positive predictive value, *NPV* negative predictive value, *K* diffusion kurtosis, *D* diffusion coefficient, *K^trans^* transfer constant from plasma to extravascular extracellular space, *K_ep_* rate constant from extravascular extracellular space to plasma, *V_e_* fractional volume of the extravascular extracellular space, *iAUC* initial area under the contrast agent concentration-time curve. Data in parentheses are the numerator and denominator used to calculate percentages. K_ep_ and K^trans^ values are expressed in min^-1^; iAUC values are expressed in mmol·s/kg. D values are expressed in ×10^-3^ mm^2^/s.

**Supplementary Material 4**

**Table E4.** Intraclass Correlation Coefficient for DKI and DCE-MRI Quantitative Parameters

| Parameters | Interobserver | Intraobserver |
| --- | --- | --- |
| DKI parameters |  |  |
| D (×10^-3^ mm^2^/s) | 0.94 (0.90, 0.96) | 0.97 (0.96, 0.98) |
| K | 0.92 (0.87, 0.95) | 0.98 (0.96, 0.99) |
| DCE-MRI parameters |  |  |
| K^trans^ (min^-1^) | 0.93 (0.90, 0.96) | 0.98 (0.97, 0.99) |
| K_ep_ (min^-1^) | 0.92 (0.87, 0.95) | 0.99 (0.98, 0.99) |
| V_e_ | 0.96 (0.93, 0.97) | 0.97 (0.94, 0.98) |
| iAUC (mmol·s/kg) | 0.92 (0.87, 0.95) | 0.92 (0.87, 0.95) |

*DKI* diffusion kurtosis imaging, *DCE-MRI* dynamic contrast enhanced MRI, *D* diffusion coefficient, *K* diffusion kurtosis, *K^trans^* transfer constant from plasma to extravascular extracellular space, *K_ep_* rate constant from extravascular extracellular space to plasma, *V_e_* fractional volume of the extravascular extracellular space, *iAUC* initial area under the contrast agent concentration-time curve. Data are intraclass correlation coefficients and data in parentheses are 95% confidence intervals.

**References**

1 Tofts P (1997) Modeling tracer kinetics in dynamic Gd-DTPA MR imaging. J Magn Reson Imaging 7:91-101

2 Orton M, d'Arcy J, Walker-Samuel S et al (2008) Computationally efficient vascular input function models for quantitative kinetic modelling using DCE-MRI. Phys Med Biol 53:1225-1239

3 Parker G, Roberts C, Macdonald A et al (2006) Experimentally-derived functional form for a population-averaged high-temporal-resolution arterial input function for dynamic contrast-enhanced MRI. Magn Reson Med 56:993-1000

4 Weinmann H, Laniado M, Mützel W (1984) Pharmacokinetics of GdDTPA/dimeglumine after intravenous injection into healthy volunteers. Physiol Chem Phys Med NMR 16:167-172
